# Fabrication and Characterization of Sulfonated Graphene Oxide-Doped Polymeric Membranes with Improved Anti-Biofouling Behavior

**DOI:** 10.3390/membranes11080563

**Published:** 2021-07-27

**Authors:** Muhammad Zahid, Anum Rashid, Saba Akram, H. M. Fayzan Shakir, Zulfiqar Ahmad Rehan, Talha Javed, Rubab Shabbir, Mahmoud M. Hessien

**Affiliations:** 1 Department of Chemistry, University of Agriculture, Faisalabad 38000, Pakistan; rmzahid@uaf.edu.pk; 2 Department of Materials, National Textile University, Faisalabad 37610, Pakistan; anumrashid800@yahoo.com (A.R.); saba.akram1980@gmail.com (S.A.); fayzan.shakir@ntu.edu.pk (H.M.F.S.); 3 College of Agriculture, Fujian Agriculture and Forestry University, Fuzhou 350002, China; mtahaj@fafu.edu.cn (T.J.); rubabshabbir28@gmail.com (R.S.); 4 Department of Agronomy, University of Agriculture, Faisalabad 38040, Pakistan; 5 Seed Science and Technology, University of Agriculture, Faisalabad 38040, Pakistan; 6 Department of Chemistry, College of Science, Taif University, P.O. Box 11099, Taif 21974, Saudi Arabia; m.hessien@tu.edu.sa

**Keywords:** cellulose acetate, sulfonated graphene oxide, nanocomposite membrane, anti-biofouling

## Abstract

In this study, cellulose acetate (CA) was blended with sulfonated graphene oxide (SGO) nanomaterials to endow a nanocomposite membrane for wastewater treatment with improved hydrophilicity and anti-biofouling behavior. The phase inversion method was employed for membrane fabrication using tetrahydrofuran (THF) as the solvent. The characteristics of CA-SGO-doped membranes were investigated through thermal analysis, contact angle, SEM, FTIR, and anti-biofouling property. Results indicated that anti-biofouling property and hydrophilicity of CA-SGO nanocomposite membranes were enhanced with addition of hydrophilic SGO nanomaterials in comparison to pristine CA membrane. FTIR analysis confirmed the successful decoration of SGO groups on CA membrane surface while revealing its morphological properties through SEM analysis. Thermal analysis performed using DSC confirmed the increase in thermal stability of CA-SGO membranes with addition of SGO content than pure CA membrane.

## 1. Introduction

Membrane technology has undergone advances in various fields of application like wastewater treatment, water purification, food, seawater desalination, and medicine, which is attributed to its advantageous features like simplicity, high efficacy, eco-friendly nature, insignificant chemical utilization, and cost effectiveness [1]. In previous years, a great focus has been on the development of polymeric materials with high performance which must possess good hydrophilicity, high permeability, and excellent separation [2]. However, the main obstacle which economically and from technical point of view restricts membrane performance is its “biofouling”.

Biofouling is caused by the deposition, attachment, and proliferation of biological foulants (e.g., proteins and bacterial cells) present in feed water on the surface of a membrane, resulting in biofilm formation. Biofouling assists in the concentration polarization (i.e., the ratio among solute concentration on the surface of membranes and inside bulk solution) of nutrients on the membrane surface [3], which consequently blocks the membrane pores, causes tremendous reduction in salt rejection, permeates flux, and increases transmembrane pressure, thus requiring further energy for filtration [4,5]. Therefore, the key focus in context of membrane modification is fabrication of anti-biofouling membranes [6]. As membrane morphology and hydrophilicity play pivotal roles in membrane-based separation processes, they can be helpful in resolving biofouling issues [7].

Cellulose acetate (CA) is the most commonly employed polymer in membrane fabrication and is known as first one to use for filtration membranes in purification of water [8]. It is a biobased material; is renewable; and possesses various reliable properties, i.e., high biocompatibility, potential flux, and moderate hydrophilicity. Besides these advantages, CA exhibits poor resistance towards fouling issues [9,10]; biofouling is more favorable on polymeric membranes of hydrophobic nature, as when the membrane comes into contact with proteins, strong hydrophobic–hydrophobic interactions occur. Therefore, improving hydrophilicity and morphological features of a membrane is a common strategy in biofouling mitigation [11].

Therefore, for enhancing hydrophilicity and anti-biofouling features of cellulose acetate membrane it is blended with hydrophilic inorganic materials to generate a nanocomposite membrane, which is recognized as a common modification strategy for polymeric membranes in recent years. Addition of nanomaterials results in considerable change in membrane properties like mechanical, magnetic, thermal, morphological, hydrophilic, and anti-biofouling in comparison to unfilled membranes [12,13,14]. Some nanoparticles commonly utilized in the modification of polymer membranes include titanium dioxide (TiO_2_), alumina (Al_2_O_3_), silica (SiO_2_), zinc oxide (ZnO), carbon nanotubes, GO nanosheets, SGO, Fe_3_O_4_, clay, and zirconia (ZrO_2_) [15,16,17,18,19]. Mostly, the issue encountered by hybrid membranes is that nanoparticles possessing low specific surface, once added into the casting solution at high concentration, result in agglomeration, which can cause imperfect pore formation of resulting membranes. Therefore, the selection of nanoparticles with high specific surface area but low additive proportion is important [20,21]. The anti-biofouling property is determined usually by antibacterial activity and the mechanism occurs during the interaction of nanoparticles with thiol group of cysteine that usually exist in membrane’s cell of bacteria [11].

Graphene oxide (GO) is a remarkable material with abundance of oxygen-containing functional groups, i.e., carbonyl, carboxyl, hydroxyl, and epoxy groups, which are hydrophilic in nature [22]. GO exhibits extremely low density, high strength, high aspect ratio, unique planar structure, and easy surface functionalization [22]. However, the amphiphilic nature of graphene oxide limits its water uptake ability (hydrophilicity) in the case of composite membranes [23], which means that hydrophobic pollutants like proteins can be absorbed upon the membrane surface. Therefore, an increase in hydrophilicity offers enhanced resistance against biofouling as most of microbes, plants, and proteins are hydrophobic in nature [24]. Another problem associated with GO is the difficulty in producing a homogenous dispersion inside the membrane matrix; due to the high nanomaterial concentration, agglomeration can easily occur, which eventually reduces the membrane performance in terms of decreased hydrophilicity and water flux. Thus, in order to achieve a membrane with high performance, it is important to decrease the nanoparticles agglomeration through attachment of various functional groups on its surface using different chemical reactions [1]. 

In the current research work, graphene oxide (GO) surface was functionalized with hydrophilic sulfonic acid groups to form a nanocomposite additive with increased hydrophilicity, anti-agglomeration, and an improved negative charge that is subsequently blended with cellulose acetate for fabrication of novel cellulose acetate-doped SGO composite membrane. The influence of SGO additives upon membrane properties, i.e., wettability, morphology, structure, and chemistry, along with membrane performance in terms of anti-biofouling behavior and water permeability, were investigated.

## 2. Materials and Methods

### 2.1. Materials

Cellulose acetate (CA) of Mw = 50 kDa in powder form was purchased from Sigma Aldrich (St. Louis, MO, USA). Before use, CA powder was dried in vacuum oven at 105 °C. Tetrahydrofuran (THF, 99.5%), sodium nitrate (NaNO_3_), ethylene glycol (99.5%), sulfuric acid (98.5%), KMNO_4_ (99.7%), graphite powder (99.99%), and NH_4_OH were purchased from Sigma-Aldrich (St. Louis, MO, USA). Ultrapure water (Pure lab Elga, High Wycombe, UK) was used for the preparation of the solutions. Unless specified, all of the reagents were used as received without further purification.

### 2.2. Preparation of Graphene Oxide (GO)

Synthesis of Graphene oxide was done using Hummer’s method. The procedure involves the following steps: 

The graphite powder (6 g) was added along with 200 mL sulfuric acid and 2.5 g sodium nitrate into flask with constant stirring. In the next step, 30 g of KMnO_4_ (Potassium Permanganate) was then slowly added into flask containing suspension for temperature maintenance up to 5 °C and kept in an ice bath with continuous agitation for 3 h. The ice bath was then removed, and the temperature of suspension increased for 45 min up to 35 °C. Distilled water (200 mL) then subsequently added into suspension with rise in temperature for 20 min up to 98 °C. The color of suspension turned brown. Distilled water (250 mL) was added for suspension dilution followed by the addition of 20 mL of H_2_O_2_ (10%) for reduction of unreacted manganese dioxide and permanganate into soluble manganese sulfate. During this process, the suspension turned bright yellow followed by centrifugation of suspension which resulted in a yellow brownish paste. The suspension was washed twice using 95% alcohol and three times with deionized water, yielding graphitic oxide. At last, we sonicated graphitic oxide to generate separate layers known as graphene oxide. The resulting GO placed in oven for drying at 60 °C for 12 h [25].

Sulfonation of GO was achieved using Diazonium salt of Sulfanilic acid. Steps involved in Diazonium salt preparation are as follows:

(a) First, sulfanilic acid was dissolved in solution of NaOH (10%) using water bath and little bit heating. (b) Then, the heated solution was allowed to cool at room temperature followed by addition of sodium nitrite (NaNO_2_). (c) The solution was then placed in an ice bath under continuous stirring followed by addition of 10 mL concentrated HCl and 10 mL cooled water until the formation of white solution. (d) Afterwards, 1 g of the GO suspension was added to 100 mL of distilled water using sonication, which was then added into the above solution of diazonium salt for 4 h with addition of few drops of hydrazine to ensure a bit reduction of GO surface. (e) The suspension so obtained using centrifugation was then washed several times with distilled water (f) Last, the sulfonated graphene oxide (SGO) was obtained [26].

### 2.3. Fabrication of SGO-Cellulose Acetate Membranes

**Figure 1:**
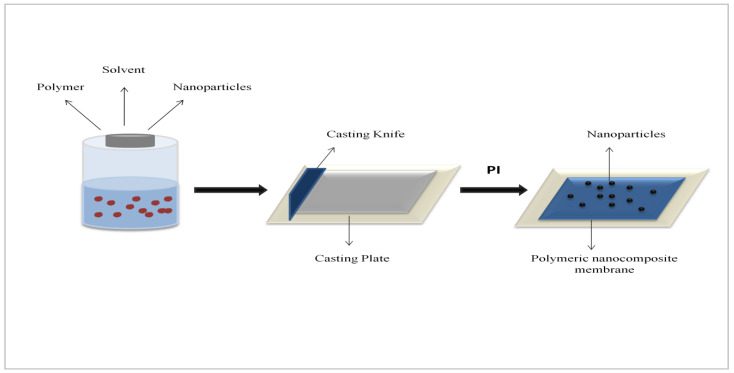
Phase inversion process for membrane fabrication.

The preparation of the SGO-blended CA membrane was done using non-solvent-induced phase inversion (NIPS) [27]. The compositions of casting solutions of CA, THF as solvent, and the desired concentration of SGO nanomaterials are given in Table 1. In the beginning, the precise SGO nanoparticle amount was poured into THF solvent and dispersed for 30 min using a sonicator (ultrasonic water bath) for improvement in homogeneity. On other side, CA was added into the THF solution and under continuous stirring was poured with the dispersion of SGO at room temperature. For better dispersion of SGO, nanomaterials into CA matrix and elimination of air bubbles the solution was kept for 24 h. The casting solution was then ready to be casted using casting glass plate with thickness of (150 μm) and casting knife, and the film so obtained was then submerged into coagulation bath containing distilled water for almost 2–3 min. Afterwards, the prepared film was detached from casting plate by washing three times using pure water. The membrane so obtained was allowed to dry at room temperature and then stored for further use (Figure 1).

### 2.4. Characterization of GO and SGO Nanoparticles and CA-Composite Membranes

FTIR spectra for GO and SGO composite membranes were taken using a Perkin Elmer FTIR spectrometer (Waltham, MA, USA). The morphological features (i.e., top surface and membrane microstructures) of the prepared membranes were characterized using SEM (FEI, Quanta FEG 450, Thermo Scientific, Waltham, MA, USA). The crystalline structure of prepared SGO doped CA membranes was characterized through X-ray diffraction (PANalytical X’pert, Malvern Panalytical, Malvern, UK) technique, respectively. 

Thermal properties of SGO doped composite membranes were determined using differential scanning calorimetry (DSC-250, TA instruments, New Castle, DE, USA) at a 10 °C/min heating rate under nitrogen flow of 30 mL/min. Ten milligram samples were sealed in an aluminum pan and scanned over a range of 25 °C to 500 °C [28]. 

### 2.5. Measurement of Water Contact Angle, Pure Water Flux, Tensile Strength

The contact angle was measured through Attension Theta Tensiometer using the sessile-drop method. The amount of water used was 5 µL. In this method, a droplet of distilled water was dropped on the membrane surface and then the contact angle of the droplet with surface was calculated. Five random locations of each membrane were selected for each measurement of the contact angle to minimize the investigation error and then the average was reported. Pure water flux of membrane was checked using a cross-filtration apparatus and mechanical properties like tensile strength of fabricated membrane were evaluated using Instron tensile testing machine. Particularly, for each type of membrane the specimens were stretched in unidirection at the rate of 5 mm/min [29]. The pure water flux of SGO membranes was determined using dead end stirred filtration system having membrane active area of ~18.5 cm^2^. The filtration cell was mounted to a reservoir containing deionized water and compressed via nitrogen gas. First, the membrane was compacted for 30 min using filtering water at 2 bar (gauge), and then filtration tests were performed at 1 bar (gauge). Permeated water was then collected and weighed out using digital balance. The following formula was employed for the calculation of pure water flux Jw (L m^−2^h^−1^) [29]:

$$Jw=\frac{V}{A\times \Delta t}$$

where V is the permeate volume (L), A is the effective area of membrane (m^2^) and Δt is the time of filtration (h).

### 2.6. Antibacterial Testing

Antibacterial testing was conducted using disc diffusion method. The inoculum of bacteria was dispersed homogeneously using nutrient agar on sterile petri dish with the help of sterile cotton swab. In this study, *Escherichia coli* pathogenic bacteria was used. For testing, each membrane punched in the shape of disks was transferred into a petri dish containing culture of grown bacteria. Afterwards, these plates were placed for incubation at 36 °C ± 1 °C for 24 h, under aerobic conditions. Then, the inhibition zone was measured at the end against each sample [25]. 

## 3. Results and Discussion

**Figure 2:**
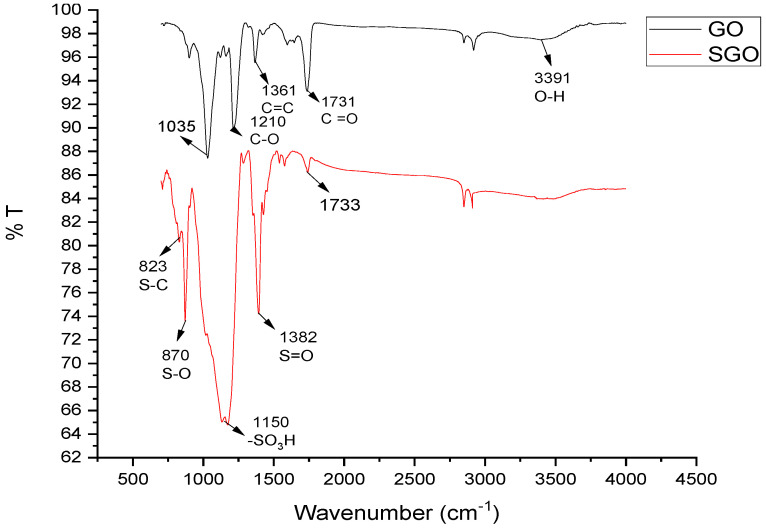
FTIR spectra of pure GO and SGO.

The analysis of sample using FTIR was done for the determination of functional groups. Graphene oxide and SGO were analyzed using this spectroscopic technique. The FTIR spectra given in Figure 2 give the comparison among sulfonated graphene oxide (SGO) and GO. GO shows its characteristic peaks at 3000–3389 cm^−1^ corresponding to hydroxyl (–OH) group and peak at 1731 cm^−1^ to carbonyl (C=O) groups, whereas at 1210, 1035 cm^−1^, 1361 cm^−1^ is related to stretching vibrations of epoxy (C–O), alkoxy (C–O) groups, and C=C aromatic bonds (un-oxidized sp^2^), respectively [26]. The peak obtained at 1150 cm^−1^ corresponds to group, i.e., of sulfonic acid [–SO_3_H], whereas it is absent in GO spectrum. The peak at 1733 cm^−1^ in spectrum is decreased as in GO, which showed that in process of sulfonation the graphite oxide is reduced partially. The sharp peaks obtained at 1204 cm^−1^ and 1382 cm^−1^ reveal asymmetric and symmetric S=O stretching modes, confirming the successful substitution of sulfonic groups upon the GO surface. The peaks obtained close to 823 cm^−1^ and 875 cm^−1^ are related to stretching modes of S–C and S–O bonds, respectively, signifying the covalent existence of –SO_3_H on the surface of reduced GO. The reduction in broad peak at 3369 cm^−1^ suggested the partial reduction of sulfonated GO [30].

**Figure 3:**
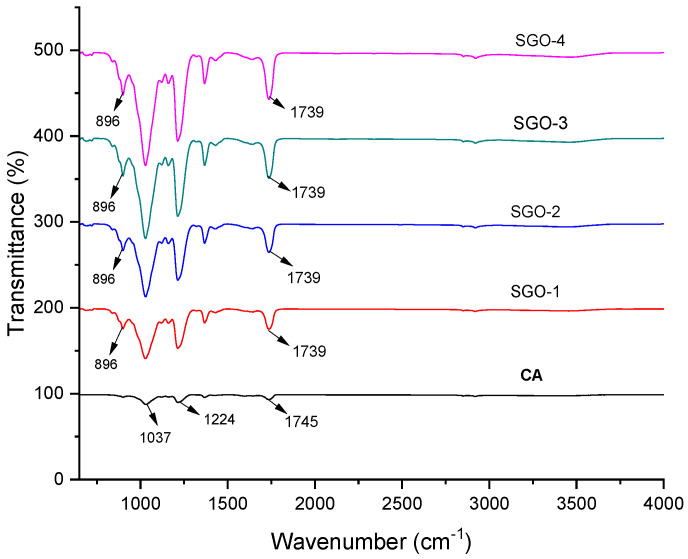
FTIR analysis of CA-doped SGO membranes.

The FTIR spectra of pristine CA and CA-SGO nanocomposite membranes at various concentrations (SGO-1 = 0.02%; SGO-2 = 0.06%; SGO-3 = 0.1%; SGO-4 = 0.14%) are shown in Figure 3. Characteristic peaks exhibited by pure CA membrane are at 1037, 1224, 1369, and 1745 cm^−1^ which corresponds to C–O cm^−1^, C–O–C cm^−1^, C–CH_3_ cm^−1^, C=O cm^−1^, and O–H cm^−1^ stretching modes of vibration. The IR absorptions frequency of pristine CA membrane is almost identical to CA–SGO nanocomposite membranes. However, the characteristic peaks exhibited by SGO at 823 and 896 cm^−1^ related to stretching mode of vibration for S–O and S–C, respectively. The presence of peak at 1739 cm^−1^ in spectrum for all SGO membranes showed that in process of sulfonation the graphite oxide is reduced partially.

**Figure 4:**
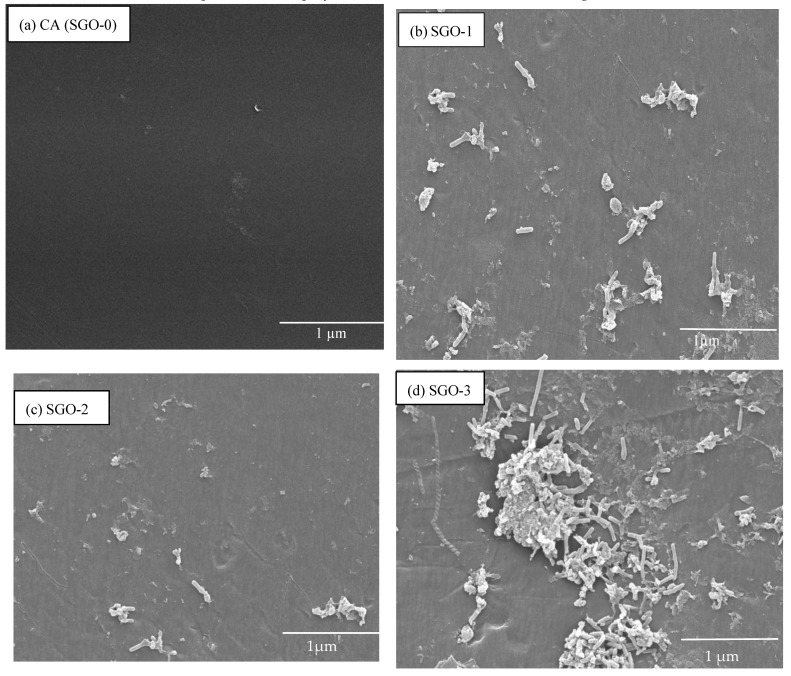
SEM images of (**a**) CA (SGO-0), (**b**) SGO-1, (**c**) SGO-2, and (**d**) SGO-3 anti-biofouling membranes.

Figure 4a–d depicts the surface morphological features of CA and composite membranes, respectively. We provide a SEM image of pure CA membrane given in Figure 4a with homogeneous surface, whereas Figure 4b–d represents cellulose acetate composite membranes with varying concentrations of SGO. SGO-1 shows membrane with less anti-biofouling behavior due to less loading of SGO particles. However, with the increase in concentration of SGO within CA membrane, the anti-biofouling behavior of the corresponding membranes enhances due to reduction in fouling layer on the membrane surface (Figure 4c,d). Furthermore, the SGO-2 and SGO-3 membranes show a smooth surface with fewer particles spreading, meaning that the addition of SGO nanofiller did not crack membrane surface at this concentration. On further increase in concentration, particles start aggregating on membrane surface at intervals which reflect incomplete particles dispersion within polymeric material and low anti-biofouling behavior [31].

**Figure 5:**
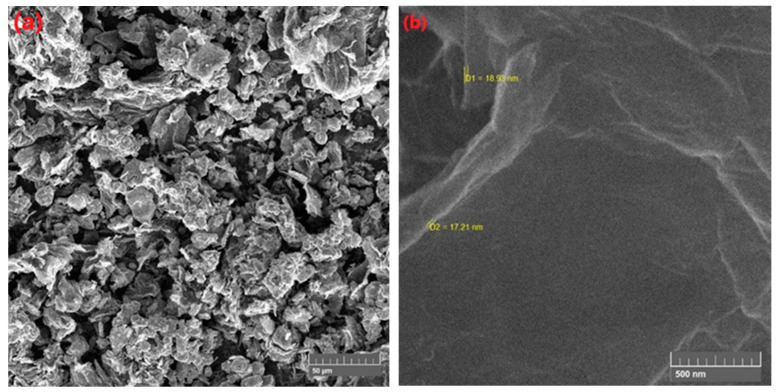
Morphological study of (**a**) SGO at 50 µm magnification and (**b**) estimated particle size.

Figure 5a,b shows the morphological features of SGO with particles sizes determined. The SEM analysis indicates the thickness in the range of 11 to 14 nm for prepared SGO nanoparticles. Moreover, particle size was also calculated through Scherrer calculator feature, which measured particle size of 6–8 nm thus confirms the fabrication of sulfonated graphene oxide [32].

**Figure 6:**
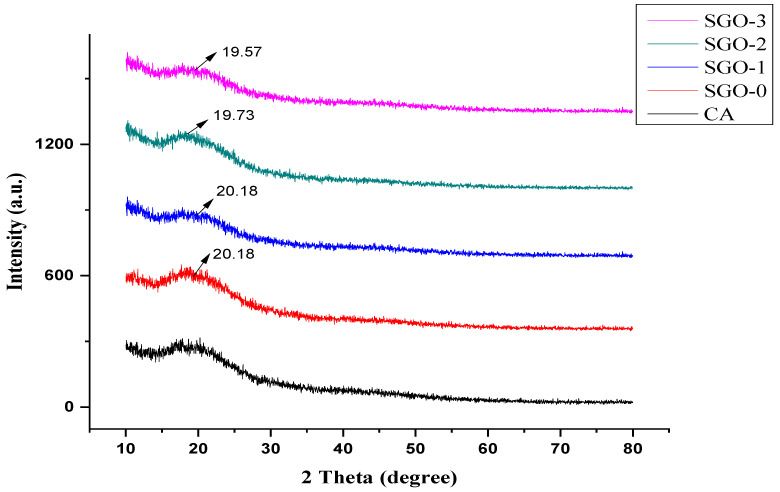
XRD analysis of CA-SGO membranes.

The crystallinity of CA-SGO nanocomposite membranes, determined by XRD analysis, reflects the halo amorphous structure as shown in Figure 6. An amorphous halo exits within the range of 15 to 30°. The existence of broader peaks at 2 theta values of 19.57 and 20.18 corresponds to GO and SGO, respectively. It suggests that all the membranes exist in an amorphous structure. Similar patterns are reported in another work conducted in [3].

**Figure 7:**
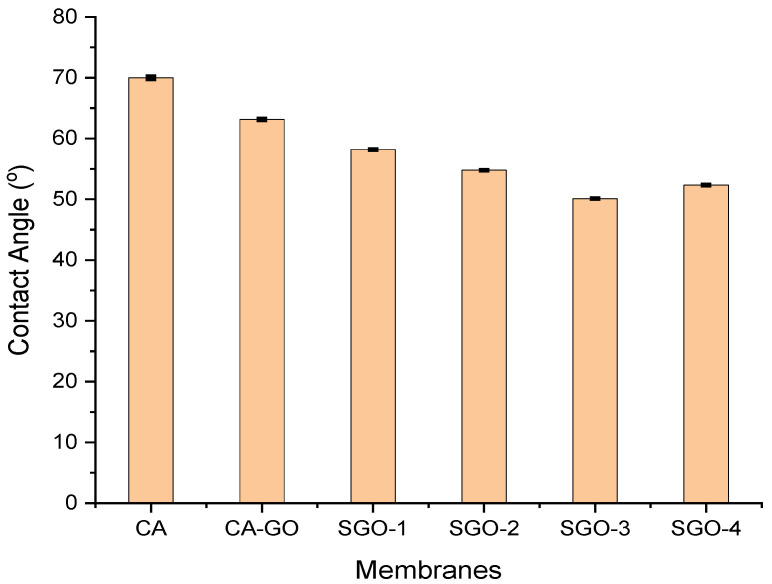
Contact angle of CA and composite membranes.

Contact angle measurement of membranes is widely used for characterizing the membrane surface hydrophilicity. One of the most significant characteristics of membranes is their hydrophilicity because it can influence the anti-biofouling ability and flux of membranes [12]. The highest contact angle of 70° is shown by CA membrane. After adding hydrophilic SGO nanofillers, a decrease in contact angle of composite membranes is observed which suggested better hydrophilicity and improved affinity of water for CA-SGO composite membranes (Figure 7). Furthermore, all CA-SGO membranes exhibited higher hydrophilicity than the CA–GO (SGO-0 membrane). This is attributed to the introduction of water holding sulfonic acid –SO_3_H, –COOH, and O–H moieties both on the surface and within the matrix of respective hybrid membranes. 

These functionalities have the ability to absorb water molecules quickly through ion-dipole and dipole–dipole interactions. Consequently, an increase in hydrophilic character of CA composite membranes will help in promoting anti-biofouling performance and membrane permeability [21,33].

**Figure 8:**
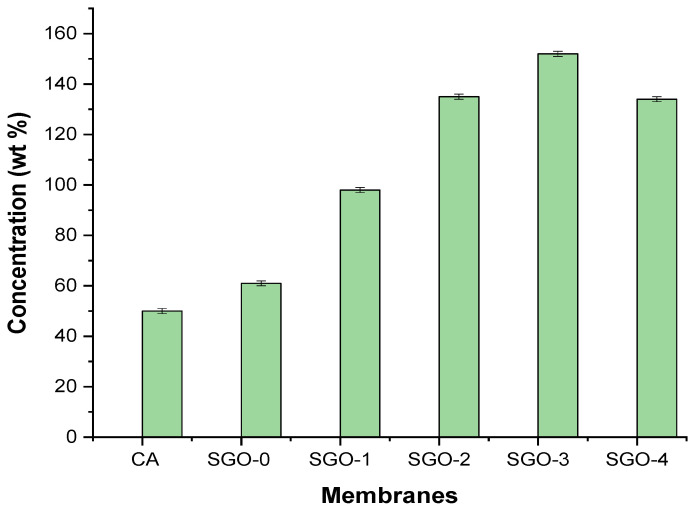
Pure water flux of CA and composite membranes.

Figure 8 shows the effect of GO and SGO nanofillers on water flux of cellulose acetate blended membranes. The results suggested that CA blended membranes with GO and SGO led to an increase in water flux as compared to pure CA membrane. The water flux for pristine CA membrane was 50 Lm^−2^h^−1^/bar. Addition of SGO (SGO-1, SGO-2, SGO-3, and SGO-4) nanofillers in the casting solution of CA improved the visible changes in the pure water flux. Among the CA-SGO blended membranes, the water flux of SGO-3 reached a peak value of 152 Lm^−2^ h^−1^/bar in comparison to pure CA membrane. This indicates that the presence of additional sulfonic groups (SO_3_H) upon graphene oxide (GO) holds a thick layer of water and hence increases the water flux. The –SO_3_H group anchored within SGO is a strong H-bonding group rather than the –OH/–COOH groups present on GO. Additionally, SGO nanofillers showed a decrease in contact angle which can positively act in promoting the permeability of water [21]. Moreover, the slight decrease in water flux is shown by high SGO-4 (1.4 wt %) content mainly due to pore blockage because of nanoparticles agglomeration at highest concentration of embedded nanoparticles within fabricated membranes.

**Figure 9:**
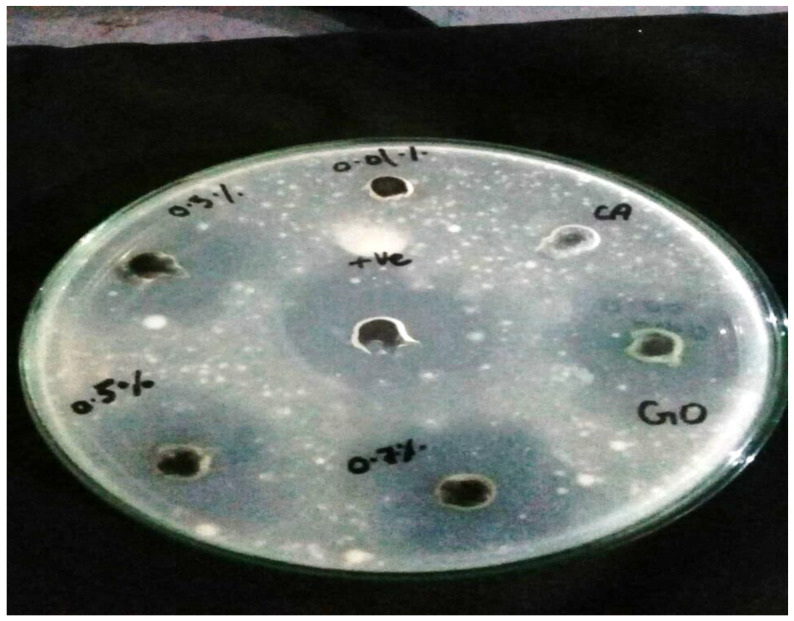
Inhibition zone measurement using agar diffusion method.

**Figure 10:**
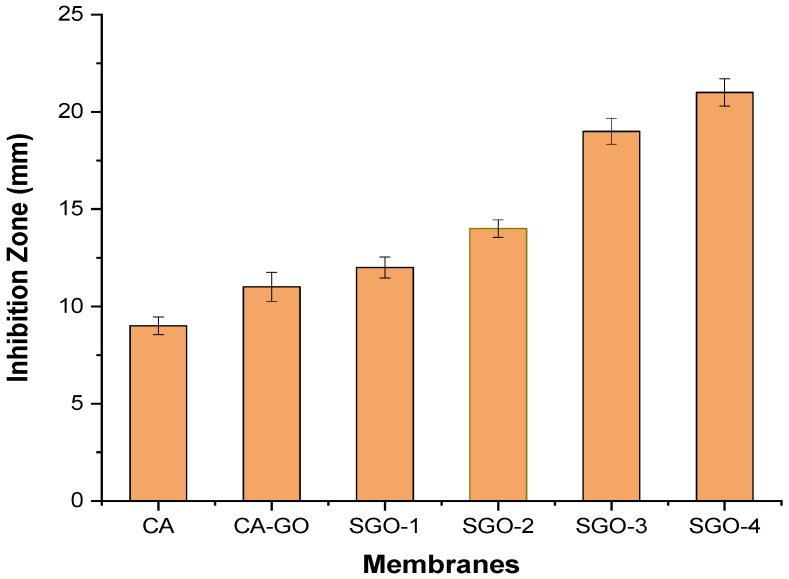
Anti-biofouling evaluation of CA and composite membranes.

The antibacterial activity of fabricated CA-based SGO-doped membranes was evaluated via agar well diffusion method through measurement of inhibition zone. Our chosen testing microbe, *Escherichia coli*, was inoculated into the plate containing agar medium through spreading over whole space. Afterwards, five to six holes of 6 to 8 mm diameter were aseptically punched with a tip or sterile cork borer into a plate containing agar medium followed by introduction of antimicrobial extract solution and control with highest antibacterial activity (*Ampicillin*) using microliter syringe into wells which already contained *E.coli* as a testing microbe. The agar plates were then incubated under suitable conditions depending on the test microorganism. This causes diffusion of antimicrobial agent into agar medium which inhibits the tested microbial strain growth. The inhibition zone was developed against each well by antimicrobial extract. From Figure 9 and Figure 10, it is revealed that antibacterial activity is increased with increased content of SGO within CA membrane. The surface of CA-SGO composite membrane exhibited negative charge due to hydrophilic SGO functional groups like sulfonic acid and hydroxyl groups which creates electrostatic repulsion among microbe and membrane [14].

**Figure 11:**
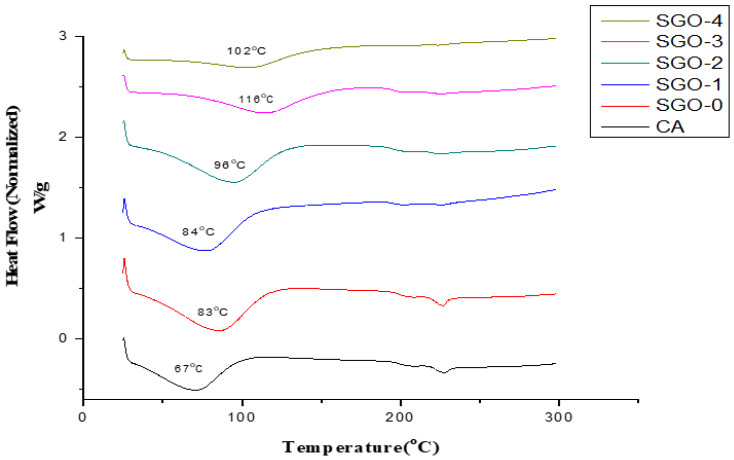
Thermal analysis of CA and CA-SGO composite membranes through DSC.

DSC curves of prepared CA/SGO membranes with different concentrations in this study are given in Figure 11. The Tg (Glass transition temperature) is generally used for interpretation of membrane structure while employing the thermal analysis upon membrane. 

It is reported that the Tg value of pure CA membrane is 55 °C [34] without any additives. The endothermic peaks shift to region of higher temperature (100–170 °C) with addition of nanofillers at higher concentration. All membranes, i.e., SGO-0, SGO-1, SGO-2, SGO-3, and SGO-4, possess a Tg value greater than 60 °C of pristine CA membrane, which confirms the enhanced thermal stability of composite membranes. A broad endothermic peak is shown generally by all membranes. This difference is attributed to differences in contents of SGO plus the density of packing among various membranes. The increase in Tg value is related to the cross-linking density of the membrane, meaning the chains of polymer are more stiff and compact [35,36].

**Figure 12:**
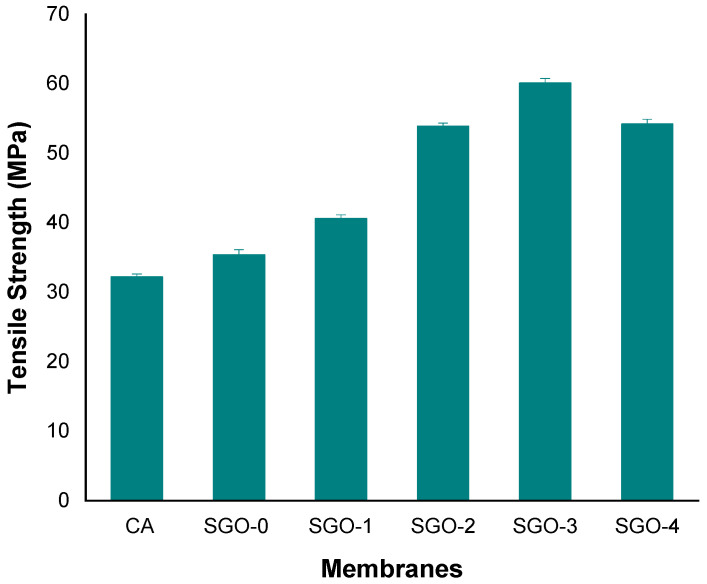
Tensile strength evaluation for CA and composite membranes.

A tensile test was used for evaluating mechanical properties of CA-SGO nanocomposite membranes. An improved in tensile strength is shown in all membranes (SGO-0, SGO-1, SGO-2, and SGO-3) as compared to pure CA membrane. As indicated from (Figure 12) due to high surface area of SGO nanoparticles an interface was obtained with CA matrix due to their better dispersion and increase in cross-linking due to SO_3_H groups presence which enhances the mechanical properties of resulting membranes. However, further increases in concentration of nanofillers, as in SGO-4, might result in decrease in tensile strength, which is related to particles aggregation on selective points of membrane during membrane fabrication.

## 4. Conclusions

In summary, CA membranes doped with SGO additives were fabricated through phase inversion method. The influence of SGO nanoparticles has been studied with main focus on the hydrophilicity, morphology, permeability, mechanical strength, and anti-biofouling behavior of CA-SGO nanocomposite membranes. With the addition of SGO nanoparticles within CA polymer matrix, an enhancement in water uptake level was observed with reduction in contact angle. SGO-3 showed maximum water flux with reduced contact angle than pristine CA membrane. The anti-biofouling behavior was enhanced through hydrophilic SGO additives which provide resistance against microbes. An increase in thermal stability was also attributed to SGO content, which efficiently reduces Tg value as compared to pure CA membrane. Based on such comprehensive results, SGO additives can be regarded as potent inorganic additives for application in wastewater treatment using various polymeric membranes.

## Figures and Tables

**Table 1:** The compositions of SGO-doped CA casting solutions.

Membrane Type	Cellulose Acetate (CA) (wt %)	Tetrahydrofuran (THF) (wt %)	Graphene Oxide (GO) (wt %)	Sulfonated GO (SGO) (wt %)
CA	15	85	-	-
SGO-0 (CA-GO)	15	84.9	0.10	-
SGO-1	15	84.98	-	0.02
SGO-2	15	84.94	-	0.06
SGO-3	15	84.9	-	0.1
SGO-4	15	84.86	-	0.14

## Data Availability

Not applicable.
