# Correction: Zahid et al. Fabrication and Characterization of Sulfonated Graphene Oxide-Doped Polymeric Membranes with Improved Anti-Biofouling Behavior. *Membranes* 2021, *11*, 563

**DOI:** 10.3390/membranes15050131

**Published:** 2025-04-29

**Authors:** Muhammad Zahid, Anum Rashid, Saba Akram, H. M. Fayzan Shakir, Zulfiqar Ahmad Rehan, Talha Javed, Rubab Shabbir, Mahmoud M. Hessien

**Affiliations:** 1 Department of Chemistry, University of Agriculture, Faisalabad 38000, Pakistan; rmzahid@uaf.edu.pk; 2 Department of Materials, National Textile University, Faisalabad 37610, Pakistan; anumrashid800@yahoo.com (A.R.); saba.akram1980@gmail.com (S.A.); fayzan.shakir@ntu.edu.pk (H.M.F.S.); 3 College of Agriculture, Fujian Agriculture and Forestry University, Fuzhou 350002, China; mtahaj@fafu.edu.cn (T.J.); rubabshabbir28@gmail.com (R.S.); 4 Department of Agronomy, University of Agriculture, Faisalabad 38040, Pakistan; 5 Seed Science and Technology, University of Agriculture, Faisalabad 38040, Pakistan; 6 Department of Chemistry, College of Science, Taif University, P.O. Box 11099, Taif 21974, Saudi Arabia; m.hessien@tu.edu.sa

## Error in Figure

In the original publication [1], there was a mistake in Figure 5a,b. The error in the published SEM image was due to an overlay issue during compilation. The corrected Figure 5a,b appears below.

The authors would like to apologize for any inconvenience caused. The authors state that the scientific conclusions are unaffected. This correction was approved by the Academic Editor. The original publication has also been updated.

## Figures and Tables

**Figure 5:**
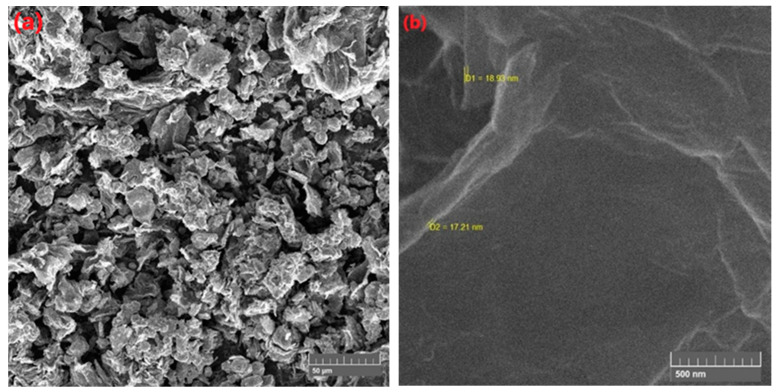
Morphological study of (**a**) SGO at 50 µm magnification and (**b**) estimated particle size.
